# Phase-Field Fracture Modelling of Thin Monolithic and Laminated Glass Plates under Quasi-Static Bending

**DOI:** 10.3390/ma13225153

**Published:** 2020-11-16

**Authors:** Jaroslav Schmidt, Alena Zemanová, Jan Zeman, Michal Šejnoha

**Affiliations:** Department of Mechanics, Faculty of Civil Engineering, Czech Technical University in Prague, Thakurova 7, 166 29 Prague 6 – Dejvice, Czech Republic; jaroslav.schmidt@fsv.cvut.cz (J.S.); Jan.Zeman@cvut.cz (J.Z.); sejnom@fsv.cvut.cz (M.Š.)

**Keywords:** laminated glass, phase-field fracture model, quasi-static bending, PVB, EVA

## Abstract

A phase-field description of brittle fracture is employed in the reported four-point bending analyses of monolithic and laminated glass plates. Our aims are: (i) to compare different phase-field fracture formulations applied to thin glass plates, (ii) to assess the consequences of the dimensional reduction of the problem and mesh density and refinement, and (iii) to validate for quasi-static loading the time-/temperature-dependent material properties we derived recently for two commonly used polymer foils made of polyvinyl butyral or ethylene-vinyl acetate. As the nonlinear response prior to fracture, typical of the widely used Bourdin–Francfort–Marigo model, can lead to a significant overestimation of the response of thin plates under bending, the numerical study investigates two additional phase-field fracture models providing the linear elastic phase of the stress-strain diagram. The typical values of the critical fracture energy and tensile strength of glass lead to a phase-field length-scale parameter that is challenging to resolve in the numerical simulations. Therefore, we show how to determine the fracture energy concerning the applied dimensional reduction and the value of the length-scale parameter relative to the thickness of the plate. The comparison shows that the phase-field models provide very good agreement with the measured stresses and resistance of laminated glass, despite the fact that only one/two cracks are localised using the quasi-static analysis, whereas multiple cracks evolve during the experiment. It was also observed that the stiffness and resistance of the partially fractured laminated glass can be well approximated using a 2D plane-stress model with initially predefined cracks, which provides a better estimation than the one-glass-layer limit.

## 1. Introduction

Glass, despite its brittleness, has proven to be a suitable material for load-bearing or fail-safe transparent structures when combined with polymers or other plastic interlayers [1,2]. Laminated glass dominates the structural applications of glass as it preserves the optical qualities of glass, and above that, it provides a certain level of post-breakage resistance [3], e.g., after an impact, an earthquake, or another disaster. Especially in the case of an unforeseen event, lamination can increase structural reliability significantly [4].

**Figure 1:**
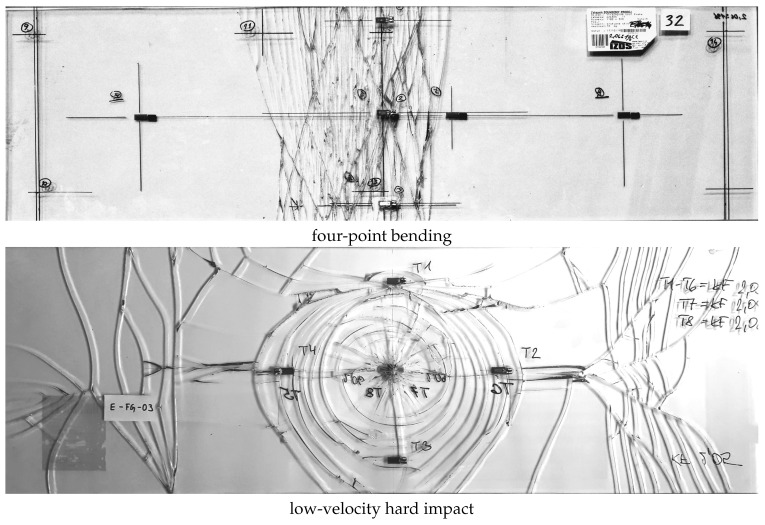
Photos of fracture patterns for laminated glass samples made of float glass with two edge supports after four-point bending with multiple cracks in the middle part or after the low-velocity hard impact of a steel impactor with a hemispherical head resulting in a spider web crack pattern.

Identifying the points of crack initiation and predicting their propagation and the subsequent response of glass elements are of paramount importance for understanding the structural performance and design of laminated glass plates. The resulting fracture pattern indicates the cause of a glass failure and therefore provides valuable information for its diagnostic interpretation (Figure 1). Different crack patterns are typical of glass plates broken due to over-stressing caused by a uniform loading, thermal stresses, a hard or soft impact, or due to a nickel inclusion or an instability failure [5].

In the literature, recent research papers in this field illustrate the effort of numerous researchers to simulate and predict the pre-fracture, fracture, and post-fracture behaviour of laminated glass. For the practical design, effective thickness approaches were derived from an analytical model to predict extreme values of deflections or tensile stresses [6,7,8,9]. Except for the methods replacing the multi-layer glass element with an equivalent monolithic one, analytical models for two plates under bending with a shear coupling provided by the interlayer were derived for laminated glass beams and plates [10,11,12,13,14,15,16]. Analytical models based on layer-wise-type theories were also tested for photovoltaic panels [17]. For numerical modelling, a comprehensive review of the finite element method in failure analyses of laminated glass can be found in [18], whereas the applicability of other numerical techniques for the impact failure analyses of laminated glass was summarised in [19].

Predicting and modelling the crack initiation and propagation in materials and structures has been one of the most significant challenges in solid mechanics for decades. In recent years, modelling based on the phase-field fracture/damage formulations has become an elegant, powerful, and well-established tool to simulate the fracture in brittle solids. The mathematical structure of phase-field formulations of fracture is similar to the continuum gradient-damage models, but their components are interpreted differently. The phase-field methods can predict crack initiation and deal with intricate crack patterns like branching. Within the scope of the widely used finite element framework, the phase-field models provide promising results for many engineering problems, e.g., [20,21,22], and became popular in the computational modelling of fracture including their recent application to glass laminates under in-plane loads [23,24] and tall laminated beams under combined in-plane tension and bending [25]. On the other hand, the phase-field models, despite their straightforward and relatively simple implementation, are computationally demanding due to the introduction of new variables in nodes corresponding to the phase-field parameters and due to the fine meshes needed.

In this paper, we focus on the pre- and post-fracture bending of laminated glass. We aim at predicting the flexural response of multi-layer samples in terms of, e.g., deflections and stresses, and discuss the corresponding fracture pattern predicted by the numerical model. This way, we want to assess the strengths and drawbacks of the phase-field formulation in fracture modelling of laminated glass under quasi-static bending and its usability for practical structural elements. Therefore, we demonstrate the performance of selected formulations on examples of thin rectangular plates of dimensions 1100 × 360 mm${}^{2}$ under bending, made of monolithic or laminated glass with two opposite sides simply supported. The presented phase-field approaches differ in the sharp crack approximation and the employed crack driving forces. Due to the fine mesh needed for the phase-field fracture analysis, different dimensional reduction strategies for the finite element discretization were utilized, i.e., multi-layer beam or plate models and a 2D sectional plane-stress model. For a fully three-dimensional numerical model, a very fine mesh would be needed for the phase-field fracture description to obtain the results with the desired accuracy. Due to the considerable numerical effort required, the approach would not be applicable to the design practice and real-sized engineering problems. Therefore, we rely on layer-wise models as an efficient tool for the analysis of multilayered plates that can provide a response comparable to 3D analyses [26,27]. For the interlayer, we tried to replace the originally viscoelastic time/temperature-dependent model [28] with an elastic one to simplify the simulations, while still obtaining reliable results.

The paper is structured as follows. Phase-field formulations tested for glass fracture are introduced in Section 2. The parameters for the numerical models are discussed and their relationships are derived in Section 3. Then, the formulations are compared for a monolithic glass plate with considering different discretizations in Section 4. The experimental program on laminated glass in bending is reviewed in Section 5, and the numerical predictions are validated in Section 6. Finally, the main conclusions from this study are summarized in Section 7.

## 2. Phase-Field Formulation for Glass Fracture

### 2.1. Overview of Selected Approaches

Phase-field damage formulations can be seen as a generalisation of Griffith’s theory of brittle fracture [29]. The formulations are based on the variational approximation [30] of brittle fracture proposed in [31]. The aim of the phase-field models is to predict the displacement field and the crack evolution by the minimisation of the total potential energy, and therefore, no other assumption for crack initiation is needed. For this purpose, a minimal set of input parameters is required, i.e., the experimentally measurable Young modulus, Poisson ratio, and critical fracture energy, plus a length-scale parameter, which can be seen as a pure numerical parameter for sharp crack regularization or as a material parameter determined under suitable assumptions from the tensile strength of the brittle material [32]. A comprehensive summary focused on the phase-field modelling of fracture can be found in [33]. The individual phase-field models proposed in the literature differ in the way the sharp crack is approximated, the degradation function of which is used to lower the stiffness with an increasing phase-field parameter, or how the irreversibility condition is enforced to avoid unphysical self-healing. These models, originally describing brittle fracture, were extended to a cohesive [34,35] and ductile [36,37] fracture or a dynamic response [38,39]. Formulations are available for one-, two-, or three-dimensional problems, for structural elements including plates and shells [40], or for multi-physics problems [41]. In this study, we compare three phase-field formulations for glass fracture:

- The classical Bourdin–Francfort–Marigo formulation with the energetic criterion (PF-B) [30,42] extended to an anisotropic model with a tension/compression asymmetry by Amor et al. [32] or Miehe et al. [43],
- The modified Miehe–Schänzel–Ulmer formulation with the stress-based Rankine-type criterion (PF-M) [44],
- The Pham–Amor–Marigo–Maurini energy-based formulation for damage evolution (PF-P) [45].

The PF-B formulation was selected for two reasons: First, it represents a widely used phase-field formulation for brittle fracture applied to engineering problems as the admissible range for the phase-field variable is intrinsically guaranteed for a quadratic degradation function. Second is to illustrate the drawback of this method in predicting the damage evolution right at the onset of loading, so the stress-strain diagram lacks an elastic phase [46].

The other two formulations, i.e., PF-M and PF-P, try to overcome the drawback of the missing elastic domain. The PF-M model replaces for this purpose the original energy-based criterion with a principal tensile stress criterion with a threshold. This formulation with a Rankine-based crack driving force becomes variationally inconsistent, but the elastic properties in uncracked zones are preserved. On the other hand, the PF-P formulation relies on the combination of a linear function for the approximation of the dissipated energy with a quadratic function for the degradation of the initial elastic energy. Then, the damage distribution in the diffuse localisation band has a parabolic shape, and the global response is linear in the initial stage. In contrast with the previous models, the admissible range for the phase-field parameter is not intrinsically satisfied, and the lower bound of this range has to be enforced by additional strategies.

Undoubtedly, many other formulations can be found in the literature. For example, alternative degradation functions, which do not yield a pronounced softening response before the fracture initiation, were systematically studied in [47].

### 2.2. Energy Functional for Elastic Bodies with Sharp Cracks

In phase-field models, the displacements u together with the cracks, represented by surfaces in the domain of the system Γ⊂Ω, are governed by the minimisation of the energy functional with respect to all admissible displacement fields $\hat{u}$ and crack surfaces $\hat{\Gamma }$, i.e.,

(1) $$\begin{array}{c} \left(u,\Gamma \right)=\underset{\left(\hat{u},\hat{\Gamma }\right)}{arg min}\left\{Ɛ\left(\hat{u},\hat{\Gamma }\right)\right\}. \end{array}$$

According to Griffith’s theory [29], this energy functional consists of three parts:

(2) $$Ɛ\left(u,\Gamma \right)=\Psi_{e}\left(u,\Gamma \right)+\Psi_{s}\left(\Gamma \right)-Ƥ\left(u\right).$$

The first term stores the elastic strain energy,

(3) $$\Psi_{e}\left(u,\Gamma \right)=\int_{\Omega \backslash \Gamma }\psi_{e}\left(\varepsilon (u)\right)\;dV,$$

where $\psi_{e}$ represents the elastic energy density with the strains defined as $\varepsilon =\nabla_{\mathrm{sym}}u$. The second term is the dissipated surface energy,

(4) $$\Psi_{s}\left(\Gamma \right)=G_{c}\int_{\Gamma }\;dA,$$

where $G_{c}$ is the Griffith-type fracture energy-per-unit-area or the critical energy release rate of the material; in what follows, the fracture energy. The last term is the functional corresponding to the work done by external forces Ƥ given by:

(5) $$Ƥ\left(u\right)=\int_{\Omega }b^{*}\cdot u\;dV+\int_{\partial \Omega_{t}}t^{*}\cdot u\;dA,$$

where $b^{*}$ are the distributed body forces in Ω and $t^{*}$ are the tractions applied on the external boundary $\partial \Omega_{t}$.

### 2.3. Phase-Field Approximation

The next step is an approximation of the sharp discontinuity with a diffuse crack. Then, the dissipated energy $\Psi_{s}\left(\Gamma \right)$ can be approximated by a volume integral:

(6) $$G_{c}\int_{\Gamma }\;dA\approx \int_{\Omega }\psi_{s}\left(d,\nabla d\right)\;dV=\frac{G_{c}}{c_{\alpha }}\int_{\Omega }\frac{1}{l_{c}}\alpha \left(d\right)+l_{c}{\left|\nabla d\right|}^{2}\;dV.$$

The phase-field variable *d* characterises the state of the material, so that d=0 corresponds to an intact material and d=1 to a fully cracked material. The geometric function α(d) characterises the evolution of the damage *d*, and the scaling parameter $c_{\alpha }$ is given by $c_{\alpha }=4\int_{0}^{1}\sqrt{\alpha (\beta )}\;d\beta$; see Table 2 on page 7. The length-scale parameter $l_{c}$ controls the width of the diffuse interface, i.e., of the continuous approximation to the discrete crack.

**Figure 2:**
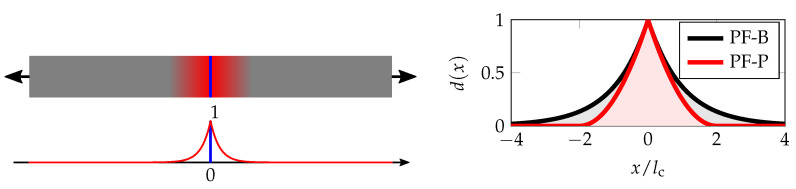
Schematic diffuse crack approximation of the originally sharp discontinuity and the detail of the damage profiles for two different geometric crack functions corresponding to a one-dimensional localised solution of an infinite bar under tension, i.e., $d=\exp(-|x|/l_{c})$ for the PF-B model or $d=(1-0.5|x|/l_{c}{)}^{2}$ for $\left|x\right|\leq 2l_{c}$ and d=0 otherwise for the PF-P model.

Examples of the one-dimensional damage profile for variationally consistent models, PF-B and PF-P, are plotted in Figure 2. If the length-scale $l_{c}$ is seen as a material parameter, its relationships with fracture energy $G_{c}$ and tensile strength $\sigma_{c}$ can be derived from a spatially homogeneous analytical solution; see [48]. For beams and plates under bending, we will discuss the setting later in Section 3.

Due to the crack regularization, the stored strain energy functional can be extended to the whole domain Ω using a degradation function g(d). Thus, the functional depends on both the displacements and phase-field variable:

(7) $$\Psi_{e}\left(u,d\right)=\int_{\Omega }g\left(d\right)\psi_{e}\left(\varepsilon (u)\right)\;dV,$$

and the corresponding energy functional reads as:

(8) $$\begin{array}{ccc} Ɛ(u,d) & = & \int_{\Omega }g\left(d\right)\psi_{e}\left(\varepsilon (u)\right)\;dV\\ & + & \frac{G_{c}}{c_{\alpha }}\int_{\Omega }\frac{1}{l_{c}}\alpha \left(d\right)+l_{c}{\left|\nabla d\right|}^{2}\;dV-\int_{\Omega }b^{*}\cdot u\;dV-\int_{\partial \Omega_{t}}t^{*}\cdot u\;dA. \end{array}$$

### 2.4. Anisotropic Formulation

Furthermore, the elastic energy needs to be decomposed into the tensile and compressive contributions, to assure that the material cracks only under tension. Two conventional approaches available in the literature are the volumetric-deviatoric split and the spectral decomposition (Table 1). The volumetric-deviatoric split is based on the degradation of the energy incorporating the positive part of the volumetric strains and the total deviatoric strains [32], whereas in the spectral decomposition, the crack evolution is induced by the positive principal strains [43].

Consequently, only the tensile part of the strain energy functional is degraded, yielding:

(9) $$\Psi_{e}\left(u,d\right)=\int_{\Omega }g\left(d\right)\psi_{e}^{+}\left(\varepsilon (u)\right)+\psi_{e}^{-}\left(\varepsilon (u)\right)\;dV.$$

Altogether, the anisotropic regularised energy function takes the form:

(10) $$\begin{array}{ccc} Ɛ(u,d) & = & \int_{\Omega }g\left(d\right)\psi_{e}^{+}\left(\varepsilon (u)\right)+\psi_{e}^{-}\left(\varepsilon (u)\right)\;dV\\ & + & \frac{G_{c}}{c_{\alpha }}\int_{\Omega }\frac{1}{l_{c}}\alpha \left(d\right)+l_{c}{\left|\nabla d\right|}^{2}\;dV-\int_{\Omega }b^{*}\cdot u\;dV-\int_{\partial \Omega_{t}}t^{*}\cdot u\;dA; \end{array}$$

compare with Equation (8). Note that the anisotropy refers here to the formulation with the tension/compression split and not to the anisotropic nature of the material.

### 2.5. Governing Equations

The unknown displacements and phase-field damage parameters are found by minimising the energy functional in Equation (10) complemented with boundary conditions. Thus, the governing equations describing the displacement sub-problem follow from Equation (10) by taking variation with respect to u,

(11) $$\begin{array}{ccc} \nabla \cdot \sigma +b^{*}=0 & & \mathrm{in}\;\Omega ,\\ \sigma \cdot n=t^{*} & & \mathrm{on}\;\partial \Omega_{t}, \end{array}$$

where n is the outward unit normal vector to the boundary ∂Ω. The stress field is given by:

(12) $$\begin{array}{c} \sigma =g\left(d\right)\frac{\partial \psi_{e}^{+}\left(\varepsilon (u)\right)}{\partial \varepsilon }+\frac{\partial \psi_{e}^{-}\left(\varepsilon (u)\right)}{\partial \varepsilon }. \end{array}$$

The phase-field sub-problem yields the damage evolution equation and the corresponding Neumann boundary condition:

(13) $$\begin{array}{ccc} \left.\begin{array}{ccc} \frac{1}{c_{\alpha }}\left(\frac{d\alpha (d)}{dd}-2l_{c}^{2}\Delta d\right)=-\frac{1}{2}\frac{dg(d)}{dd}\tilde{Y} & & \dot{d}>0\\ \frac{1}{c_{\alpha }}\left(\frac{d\alpha (d)}{dd}-2l_{c}^{2}\Delta d\right)>-\frac{1}{2}\frac{dg(d)}{dd}\tilde{Y} & & \dot{d}=0 \end{array}\right\} & & \mathrm{in}\;\Omega ,\\ \left.\begin{array}{ccc} \frac{G_{c}}{c_{\alpha }}2l_{c}\nabla d\cdot n=0 & & \dot{d}>0\\ \frac{G_{c}}{c_{\alpha }}2l_{c}\nabla d\cdot n>0 & & \dot{d}=0 \end{array}\right\} & & \mathrm{on}\;\partial \Omega , \end{array}$$

where Δ is the Laplace operator, $\dot{d}$ denotes the damage rate, i.e., the derivative of the phase-field variable with respect to a (pseudo-)time, and $\tilde{Y}$ is a normalized effective damage/crack driving force.

### 2.6. Specification of Selected Approaches

Different damage models can be derived from this general formulation by a different choice of the geometric function α and the degradation function *g* or by modification of the normalized effective crack driving force $\tilde{Y}$, as shown in Table 2.

In the PF-M formulation, the normalized effective driving force $\tilde{Y}$ in Equation (13) is replaced by a Rankine-based one in terms of the principal effective stresses (Table 2 and [44]), with the effective stress tensor given by:

(14) $$\begin{array}{c} \bar{\sigma }=\frac{\partial \psi_{e}\left(\varepsilon (u)\right)}{\partial \varepsilon }=\frac{\partial \psi_{e}^{+}\left(\varepsilon (u)\right)}{\partial \varepsilon }+\frac{\partial \psi_{e}^{-}\left(\varepsilon (u)\right)}{\partial \varepsilon }; \end{array}$$

compare with the stress tensor in Equation (12). The PF-M model is variationally inconsistent, but it can capture the asymmetric cracking behaviour with the initial linear elastic phase.

### 2.7. Staggered Scheme and Hybrid Formulation

The system of Equations (11) and (13) can be solved using a staggered solution scheme based on the alternating minimisation method, e.g., [30,49]. For this iterative scheme, the nodal displacements are solved first using the crack phase-field damage fixed from the previous iterative step. Next, the updated displacements are utilized to solve for the phase-field variable; see Algorithm 1 on page 10.

Due to the tension/compression split of the strain energy discussed in Section 2.4, the introduced anisotropic formulation leads to nonlinear equilibrium Equation (11) being solved numerically. To overcome this drawback and reduce the computational cost, a hybrid (isotropic-anisotropic) model was introduced in [50]. In this approach, the stress in Equation (12) is replaced by a term:

(15) $$\begin{array}{c} \sigma =g\left(d\right)\frac{\partial \psi_{e}\left(\varepsilon (u)\right)}{\partial \varepsilon }, \end{array}$$

that depends linearly on u; cf. Equation (8). However, the phase-field evolution is still driven by the normalized driving forces $\tilde{Y}$ in Equation (13), to avoid cracks in zones under compression. See page 10 and compare Algorithms 1 and 2.

To conclude this section, let us highlight our assumption that the phase-field model for fracture is applied to glass layers only, whereas no damage initiates and evolves in the interlayer foils. Furthermore, no delamination on the glass/polymer interfaces is allowed, so we focus purely on the glass fracture in the multi-layer laminated glass plates.

## 3. Parameters for the Phase-Field Models and Their Relation

For the numerical analysis, we present in this section material parameters needed to predict the crack initiation and propagation in glass layers. The elastic properties and tensile strength of glass in Table 3 were set according to standards [51]. The phase-field fracture formulation contains two additional parameters, which have to be specified, i.e., the fracture energy $G_{c}$ and the length-scale parameter $l_{c}$, Equation (10).

A typical value of the fracture energy $G_{c}=4$ J·m${}^{-2}$ for soda-lime-silica glass can be found in the literature [52,53]. Then, the corresponding length-scale parameter $l_{c}$, seen as a material parameter, can be estimated. For illustration, we adopt the following analytical expressions (see Table 4):

for the PF-B model [42,48,54]
  (16) $$\begin{array}{cc} l_{c} & =\frac{27}{256}\frac{EG_{c}}{\sigma_{c}^{2}}, \end{array}$$
for the PF-P model [33,55]
  (17) $$\begin{array}{cc} l_{c} & =\frac{3}{8}\frac{EG_{c}}{\sigma_{c}^{2}}, \end{array}$$
  derived from spatially homogeneous solutions of the one-dimensional quasi-static problems. This results in a very small length-scale parameter of about 10 μm and, therefore, leads to a very fine mesh and excessive computational costs for our real-sized samples or glass panels in structural glass façades. For this reason, we decided to treat the length-scale as a numerical parameter and to derive the corresponding fracture energy from Equations (16) and (17). For example, the fracture energies corresponding to the length-scale parameter of 3 mm can be seen in Table 4. This way, the length-scale parameter can be set according to the mesh density, and the fracture energy can be adjusted for the simulation.

Although derived for pure tension, the relationships introduced in Table 4 could also be used for thin structures in bending provided the stress distribution is close to constant within the finite elements in which the glass fracture could occur. Therefore, this approximation was used in the 2D plane-stress model of the longitudinal cross-section where the mesh was refined in the area of maximal bending moments.

On the contrary, the introduced relations in Equations (16) and (17) are not applicable if we employ beam and plate formulations with one element per the layer thickness characterized by a through-the-thickness constant damage parameter. To illustrate this fact, we establish relations between the length-scale parameter $l_{c}$ and the fracture energy $G_{c}$ for beams under pure bending.

Similar to previous approaches, e.g., [39,48,54], we start from the evolution equation for the damage distribution Equation (13) with $\dot{d}>0$ and neglect all spatial derivatives of *d*, as we consider the spatially homogeneous solution. By performing integration over the thickness *h*, we get:

(18) $$\begin{array}{c} \frac{G_{c}h}{c_{\alpha }l_{c}}\frac{d\alpha (d)}{dd}=2\left(1-d\right)\int_{-h/2}^{h/2}\psi_{e}^{+}\;dz. \end{array}$$

For simplicity, we limit our attention to the PF-P model. To that end, we assume that the damage starts to evolve if the largest tensile stress reaches the tensile strength $\sigma_{c}$. This allows us to write the integral on the right-hand side of Equation (18) in terms of $\sigma_{c}$ as:

(19) $$\begin{array}{c} \int_{-h/2}^{h/2}\psi_{e}^{+}\;dz=\frac{\sigma_{c}^{2}h}{12E}. \end{array}$$

If we set d=0 at the onset of cracking, Equations (18) and (19) lead for $c_{\alpha }=8/3$ and $\frac{d\alpha (d)}{dd}=1$ (recall Table 2) to the approximation of the length-scale parameter in the form:

(20) $$\begin{array}{c} l_{c}=6\left(\frac{3}{8}\frac{G_{c}E}{\sigma_{c}^{2}}\right). \end{array}$$

The same result can be derived under some simplifying assumptions for the PF-B formulation,

(21) $$\begin{array}{c} l_{c}=6\left(\frac{27}{256}\frac{G_{c}E}{\sigma_{c}^{2}}\right). \end{array}$$

## 4. Numerical Case Study of a Monolithic Glass Plate under Bending

This first part of our study is devoted to monolithic glass plates only. This way, we want to separate the effect of polymer foils and focus purely on glass response. The response of laminated glass is discussed, and the numerical model is validated against experimental data in the next two sections. The computational library FEniCS [56] was used to implement the presented models; the source codes are available in the GitLab repository [57], together with the reported experimental data. Other successful applications of the phase-field fracture in the framework of FEniCS can be found in [58,59]. All simulation results can be reproduced on conventional laptops and desktops.

Our implementation strategy for the anisotropic and hybrid staggered approach is outlined in Algorithms 1 and 2, respectively. For both algorithms, we update the phase-field parameter with the FEniCS-SNES solver, based on a semi-smooth Newton method for variational inequalities, to ensure that the parameter *d* cannot decrease in time and stays within the admissible $\langle 0,1\rangle$ interval. Additionally, the iterations with a time-step are terminated using the energy convergence control with a tolerance of $\xi_{\mathrm{SA}}=10^{-6}$. Notice that the displacement sub-problem in the anisotropic staggered scheme (Algorithm 1) involves iterative energy minimisation using the Newton method with the relative tolerance $\xi_{\mathrm{NM}}=10^{-11}$, whereas the same step in Algorithm 2 results in a linear problem; recall the discussion in Section 2.7.

**Figure 3:**
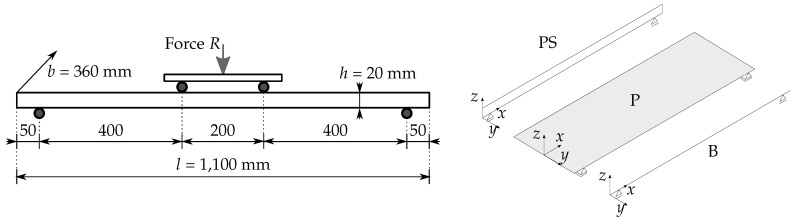
Four-point bending loading scheme and geometry of a glass sample and three different spatially reduced models, i.e., 2D plane-stress (PS) longitudinal cross-section, Reissner–Mindlin plate (P), and Timoshenko beam (B).

**Figure 4:**
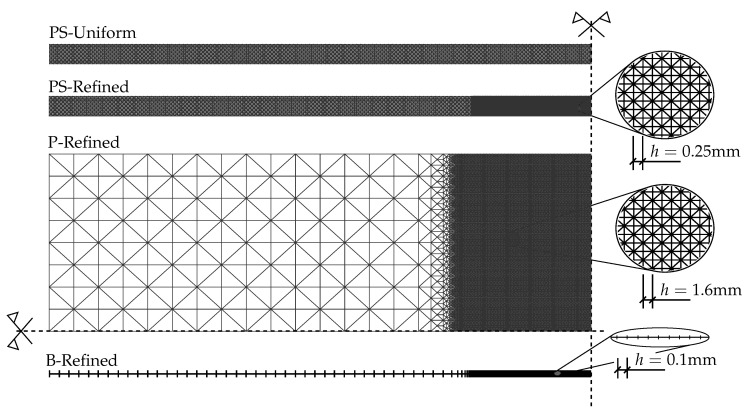
Regular uniform or refined discretization for the 2D plane-stress model (PS) and refined meshes used for a quarter of a plate (P) and a half of a beam (B).

The numerical experiments were performed on a single glass sheet of dimensions 1100 mm × 360 mm × 20 mm loaded in four-point bending; see Figure 3. The prescribed displacement of the loading head was gradually increased until fracture with the loading rate of 0.03 mm/s. The pseudo-time increment in Algorithms 1 and 2 started initially at 0.1 s and was subsequently refined to 0.01 s and finally to 0.001 s close to localisation. Regarding the discretization, we tested three different spatially reduced models schematically shown in Figure 3, i.e., the 2D plane-stress model (PS) of the longitudinal cross-section, the 2D model using the Mindlin–Reissner plate theory (P), and the 1D model based on the Timoshenko beam theory (B). For the simulations, a few types of meshes were tested, i.e., PS-Uniform: a regular uniform mesh with an element size of 2 mm, PS-Refined mesh, P-Refined mesh, or B-Refined mesh refined in the largest bending moment area (Figure 4). Let us highlight that all simulations reported in the following assume the symmetries shown in Figure 4. Hence, the simulation results and the localised crack in particular must be interpreted by taking these symmetries into consideration.

Finite elements with linear basis functions were employed in this numerical study as the phase-field formulations require relatively fine meshes in any case. Prior to modelling damage, we tested the convergence of the solution to the linear elastic problem without glass fracture. For example, for the plane-stress formulation, the errors in displacements and stresses were under 2.5% for the uniform mesh and under 1.5% for the refined variant, compared to the reference solution corresponding to the mesh of 1100 × 20 quadratic elements.

The length-scale parameter $l_{c}$ was set according to the element size, i.e., $l_{c}\approx 2h_{\min}$ with the smallest element size $h_{\min}$; see [43]. Subsequently, the corresponding fracture energy $G_{c}$ was derived according to Equations (16) and (17), or according to the modified version for beams in Equation (20) or (21).
**Algorithm 1:**Anisotropic staggered approach.
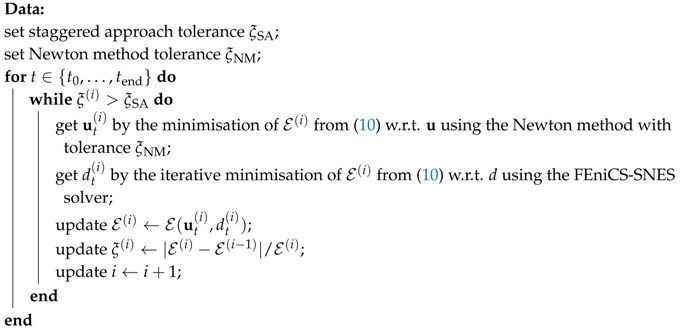

**Algorithm 2:**Hybrid (isotropic-anisotropic) staggered approach.
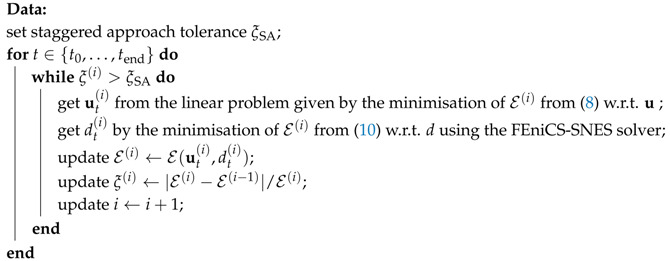


### 4.1. Effect of the Phase-Field Formulation and Mesh Refinement

**Figure 5:**
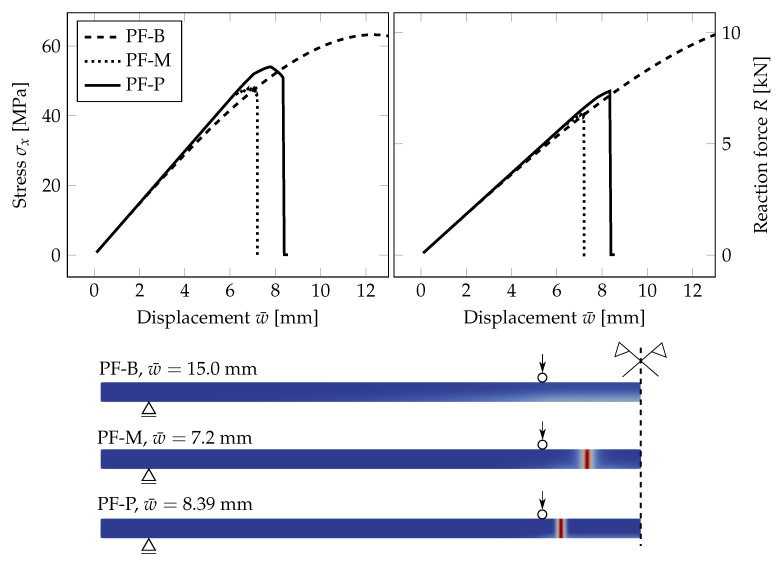
PS-Uniform mesh, anisotropic staggered solver with the spectral-decomposition split: Comparison of phase-field formulations in terms of the evolution of the largest tensile stress at the midpoint and the overall reaction under the loading points for the prescribed displacement, complemented with the damage evolution plot showing the position of the localised cracks (red).

**Figure 6:**
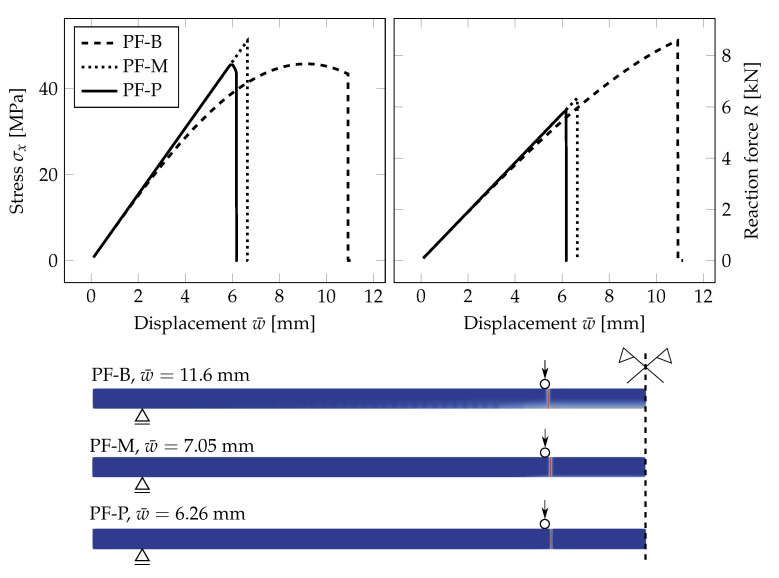
PS-Refined mesh, anisotropic staggered solver with the spectral-decomposition split: Comparison of phase-field formulations in terms of the evolution of the largest tensile stress at the midpoint and the overall reaction under the loading points for the prescribed displacement, complemented with the damage evolution plot showing the position of the localised cracks (red).

First, we present a comparison of three responses corresponding to the individual phase-field formulations using the PS model. In Figure 5 and Figure 6, the diagrams show the evolution of the largest tensile stress $\sigma_{x}$ at the midpoint and the overall reaction *R* under the loading points for the prescribed vertical displacement of the loading head $\bar{w}$, (note that the arrow represents the loading, and the triangle marks the position of the cylindrical supports). It can be seen in Figure 5 that the tensile stress evolutions differ quite significantly for the regular mesh. The PF-B model yields a nonlinear stress-strain response from the beginning of the loading test, so no initial linear phase is visible in the plots in Figure 5. Moreover, the failure stress differs for the three formulations. We attribute this discrepancy to a relatively coarse mesh and linear basis functions for the displacement field yielding constant stresses and strain energy density within an element. Consequently, the fracture occurs at different prescribed loading levels.

Therefore, we refined the mesh in the area of the largest bending moment, where the cracks are supposed to initiate. The element size in this area is about 0.25 mm; see Figure 4. The evolution of the normal stress and reaction and the damage after the crack localisation are shown in Figure 6. In this case, the failure stresses are closer to each other and to the adopted tensile strength of glass. We assume that for an even finer mesh, the performance of the PF-M and PF-P models would be almost indistinguishable. On the other hand, a significant nonlinear response prior to fracture can be seen for the PF-B formulation. Furthermore, the crack was localised in this particular case for an almost two-times higher prescribed displacement.

**Figure 7:**
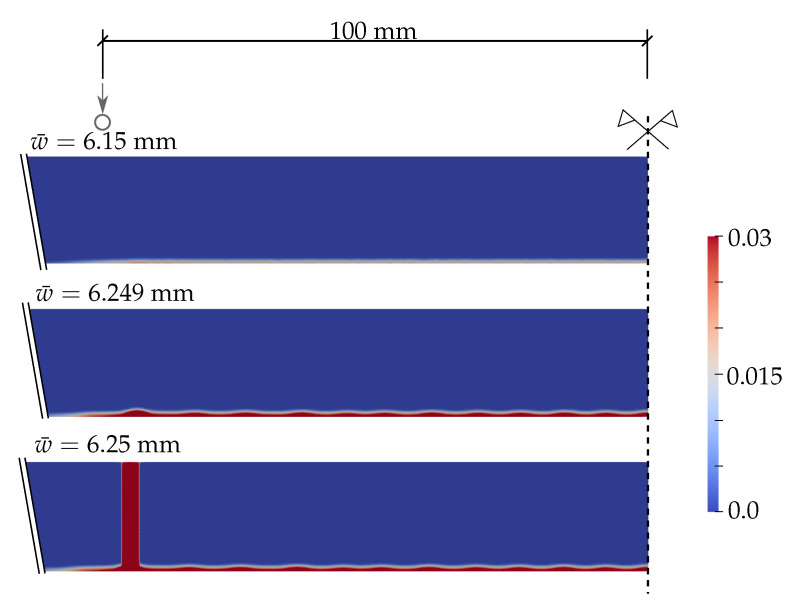
PS-Refined mesh, anisotropic staggered solver with the spectral-decomposition split: Evolution of the phase-field parameter using the PF-P formulation.

Further, Figure 7 shows the phase-field parameter evolution before the damage is localised in one crack for the PF-P formulation. We use one scale for all the plots. Initially, the damage starts to evolve in the area of the largest bending moment. Then, a few relatively equidistant short ridges appear, and finally, one crack, corresponding to the maximum of the phase-field variable, is localised close to the loading point. Hence, the phase-field model seems to predict the initiation of multiple regularly spaced cracks, from which only one is localised, as the localised solution corresponds to a lower energy value, e.g., [60,61,62].

Based on this comparison, we decided to prefer the PF-P formulation for the next analyses as it provides the initial linear elastic response of glass and preserves the variational structure of the problem. The refined mesh discretisations were used for all the following examples in this numerical study and all three spatially reduced models; the label “Refined” is omitted to shorten the notation.

### 4.2. Effect of the Type of the Solver and Tension/Compression Energy Split

Subsequently, we briefly compare the anisotropic and hybrid formulation (Section 2.7), within the staggered scheme for our example, combined with two different ways for the tension-compression split of the strain tensor from Section 2.4, i.e., the volumetric-deviatoric split and the spectral decomposition.

**Figure 8:**
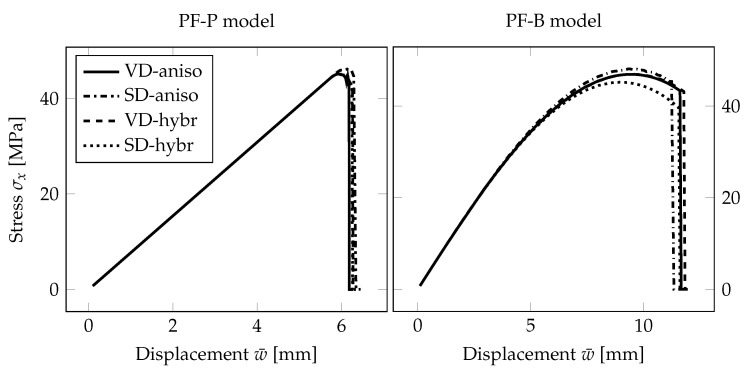
PS-Refined mesh: Comparison of the anisotropic solver, Algorithm 1, employing the volumetric-deviatoric split (VD-aniso) or spectral decomposition approach (SD-aniso) with their counterparts using a linear hybrid formulation (VD-hybr and SD-hybr), Algorithm 2.

As can be seen from Figure 8, both ways of the formulation and decomposition deliver almost identical response for the PF-P model of a thin glass plate under bending. For completeness, we show the same comparison also for the PF-B formulation. The observed differences can be attributed to the predicted early-stage degradation. However, the effect of the choice of the fully anisotropic or hybrid formulation and the split is negligible for our purposes.

### 4.3. Effect of the Dimensional Reduction

Before comparing the fracture response corresponding to different dimensional reductions, we illustrate the effect of the relationship between the fracture energy $G_{c}$ set according to Equations (17) and (20) for the selected $l_{c}$ and $\sigma_{c}$.

**Figure 9:**
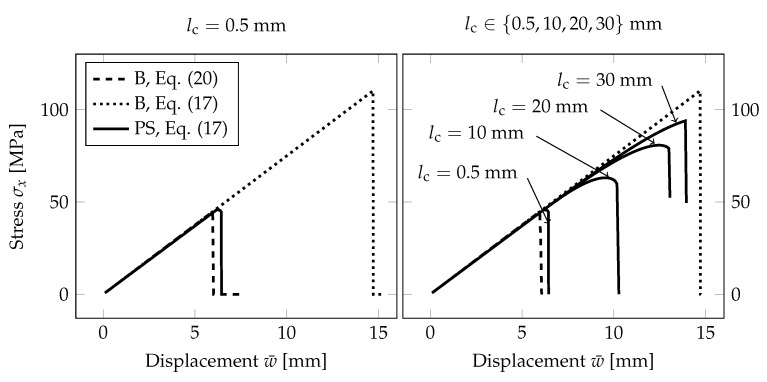
PF-P model, anisotropic staggered approach with the spectral-decomposition split: Comparison of the plane-stress model (PS) with the formulation derived for beams (B) using two constitutive relationships for $G_{c}$, $l_{c}$, and $\sigma_{c}$, i.e., $G_{c}/l_{c}=8\sigma_{c}^{2}/\left(3E\right)$ from Equation (17) and $6G_{c}/l_{c}=8\sigma_{c}^{2}/\left(3E\right)$ from Equation (20.)

Figure 9 shows the evolution of the largest tensile stress at the midpoint of the span for the increasing prescribed deflection. The PF-P formulation with the spectral decomposition was used within the staggered solver. The derived fracture energy $G_{c}$ corresponds to the critical stress $\sigma_{c}=45$ MPa and the length-scale parameter for the PS model set to $l_{c}=0.025h=0.5$ mm, i.e., twice the element size, or $l_{c}\in \left\{10,20,30\right\}$ mm, i.e., $l_{c}\in \left\{0.5h,h,1.5h\right\}$. For the B model, the length-scale parameter was fixed to the value $l_{c}=0.2$ mm, i.e., twice the element size, as its change does not affect the results in contrast with the PS model.

For the beam formulation with the mesh density shown in Figure 4, the largest failure stress at the bottom surface under tension is more than doubled compared to the given critical stress $\sigma_{c}$ with $l_{c}$ provided by Equation (17). The adjusted fracture energy according to Equation (20) provides a response comparable to the plane-stress formulation, and the fracture occurs close to the prescribed tensile strength if the length-scale parameter is much smaller than the thickness of the glass layer, e.g., $l_{c}=0.025h=0.5$ mm; see Figure 9. On the other hand, if the crack is more diffused, the failure stress on the bottom surface increases. Then, a nonlinear response can also be seen in the stress-displacement diagram for the PS model. If the length-scale parameter is greater than the glass thickness, the response of the PS model approaches the upper bound given by the beam theory using the fracture energy derived from Equation (17).

This comparison illustrates that the fracture energy has to be set with respect to the applied dimensional reduction, loading type, and the value of the length-scale parameter compared with the thickness of the structure under bending. For example, in [40], the authors reported the same response of their phase-field fracture model for plates and the reference solution for a solid using a constant $G_{c}$ value and the length-scale parameter $l_{c}$ equal to the plate thickness. However, this identical response cannot be achieved for any values of the length-scale parameter.

**Figure 10:**
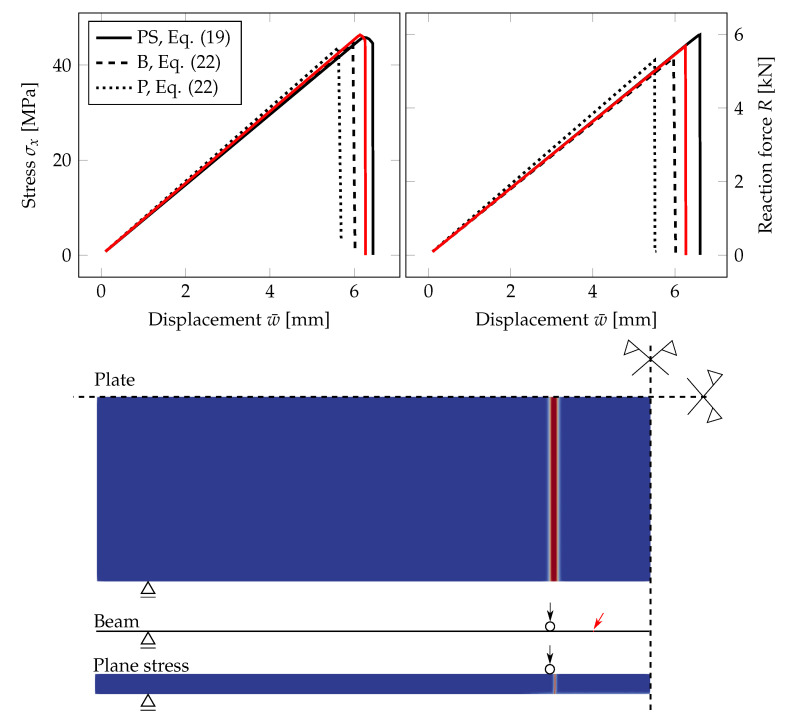
PF-P formulation, anisotropic staggered approach with the spectral-decomposition split: Comparison of plane stress (PS) for the PS-Refined mesh from Figure 4 (black line) or additionally refined in the area of the expected crack propagation (red line), beam (B), and plate (P) model in terms of the evolution of the largest tensile stress at the midpoint, the overall reaction, and the damage evolution showing the position of the localised cracks.

Figure 10 compares the response of the PS, B, and P model for a glass monolith simply supported on two sides. The evolution of the largest tensile stress and the overall reaction force *R* under the loading points for the prescribed deflection is plotted for the three models. The failure stress is almost the same for the beam and plate formulations and slightly higher for the PS model. The stiffnesses of the glass monolith corresponding to the beam theory and the PS model are equivalent, but the fracture occurs later for the PS model. The red line corresponds to a mesh additionally refined in the area of the expected crack propagation to illustrate how the responses for the B a PS models converge if the PS mesh is refined.

On the other hand, the plate formulation is stiffer, and the glass fractured for a lower prescribed deflection. We attribute these small differences again to a rather coarse mesh for the plate in some unfractured regions and to linear basis functions used in this analysis. We expect the differences to decrease at the cost of higher computational demands. The crack appeared at the same position near the loading for the PS and P model; see Figure 10. For the B model, the position of fracture is different, but still at the region of the constant largest bending moment.

**Figure 11:**
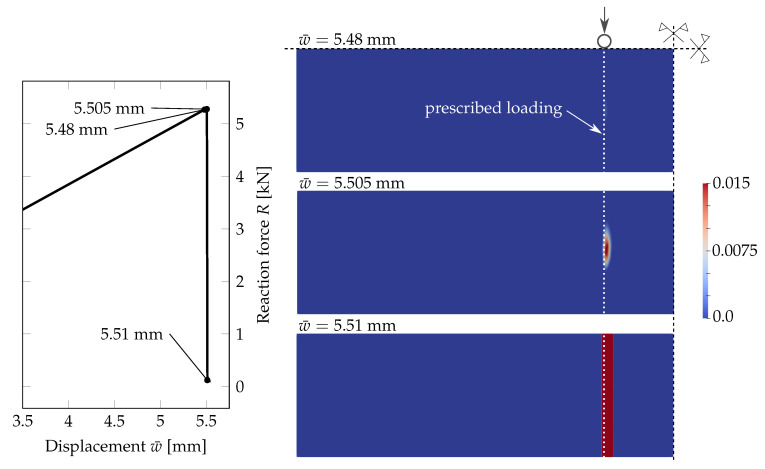
PF-P formulation, anisotropic staggered approach with the spectral-decomposition split: Evolution of the phase-field parameter on a quarter of the glass plate.

For the plate model, the evolution of the phase-field parameter is displayed in Figure 11. Each rectangle represents a quarter of the glass plate for a different magnitude of the prescribed loading. The damage starts to initiate at about a quarter of the plate width near the loading point and subsequently localises into a straight crack.

### 4.4. Reduced Glass Strength Near Plate Edges

**Figure 12:**
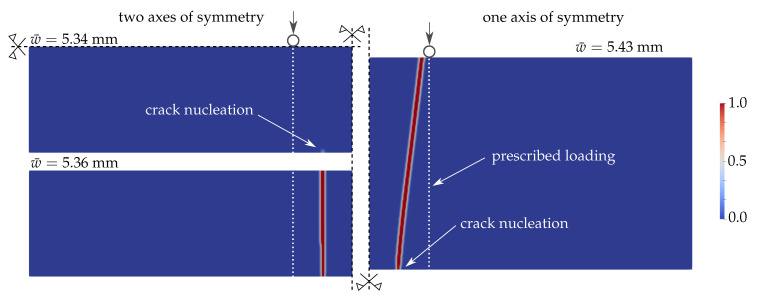
PF-P formulation, P mesh, anisotropic staggered approach with the spectral-decomposition split: Evolution of the phase-field parameter on a quarter of the glass plate and the final crack pattern on a half of the plate.

The tensile strength of thin glass plates is highly affected by micro-defects and scratches induced during the production, transport, and handling. Moreover, the edge strength depends on the quality of edge finishing. Because the region of the largest tensile stresses includes parts of both edges for the four-point bending tests, the crack mostly initiates from a spot on the edge. As suggested for example in the German glass standard [63], the strength on edges can be reduced to initiate the cracking from the edge. Near the bottom edge about 50 mm from the midspan, we modified the strength to 80% of the given tensile strength in a $1.5l_{c}\times 1.5l_{c}$ square, i.e., 4.8mm×4.8mm in our example. Then, the crack starts to initiate from this predefined area toward the opposite edge, as can be seen in Figure 12. For a smaller area, e.g., $l_{c}\times l_{c}$, the localisation does not occur from an edge point. If we use a quarter of the plate with two axes of symmetry, the final crack is almost perpendicular to the long edges as the strength was reduced on two opposite sides. On the other hand, the crack is inclined when one half of the laminated glass plate is used in the simulation (Figure 12).

Similarly, the strength can be reduced along the whole edge. The lower strength has to be assigned not only to the nodes directly on the edge, but for a band of nodes to overcome the cracking initiated on the inner surface. However, we are unable to reproduce the typical V-shaped fracture patterns, see ahead to Figure 14 on page 20, with the quasi-static plate model considering homogeneous material data in the plate interior; only one crack resulted from all numerical simulations.

## 5. Experimental Testing on Laminated Glass

The material parameters needed for the numerical model were obtained from both the literature review and experimental testing on three-layer laminated glass samples presented in this section.

### 5.1. Material Composition of Laminated Glass

Two types of laminated glass samples were tested under quasi-static loading. All of them were three-layer plates with a polymer interlayer and two glass layers of the same type. The material specification is summarised in Table 5. The nominal dimensions of samples were as follows: the length l=1.1 m, the width b=0.36 m, and the thicknesses of layers $h_{1}/h_{2}/h_{3}=10/0.76/10$ mm.

### 5.2. Testing of Polymers and the Material Model

In the present study, we exploit the results of our extensive experimental program reported in [28]. Therein, small three-layer cylindrical samples were loaded in dynamic torsion or dynamic single-lap shear under different ambient temperatures and applied frequencies. Subsequently, a set of parameters of the generalised Maxwell model for each interlayer was identified. For both polymer foils, the series employed in the numerical modelling are summarised in Table 6.

To simplify the formulation, we assume that the time-/temperature-dependent response of the interlayer can be approximated by an equivalent elastic material with the shear modulus *G* in the middle of each time interval *t* for the temperature *T*; see, e.g., [64]. Then, the shear modulus of the interlayer is evaluated in each time instant according to:

(22) $$G\left(t,T\right)\approx G_{\infty }+\sum_{p=1}^{P}G_{p}\exp^{\frac{-t/2}{a_{T}\left(T\right)\tau_{p}}},$$

with the Prony series ${\left(G_{p},\tau_{p}\right)}_{p=1..P}$ and the long-term shear modulus $G_{\infty }$ from Table 6. For the shift parameter $a_{T}$ reflecting the temperature dependency of the polymer interlayers, we employed the Williams–Landel–Ferry equation [65]:

(23) $$\log a_{T}\left(T\right)=\frac{-C_{1}\left(T-T_{0}\right)}{C_{2}+T-T_{0}}.$$

The model parameters $C_{1}$ and $C_{2}$ associated with the reference temperature $T_{0}$ are listed in Table 7.

### 5.3. Quasi-Static Bending Tests

**Figure 13:**
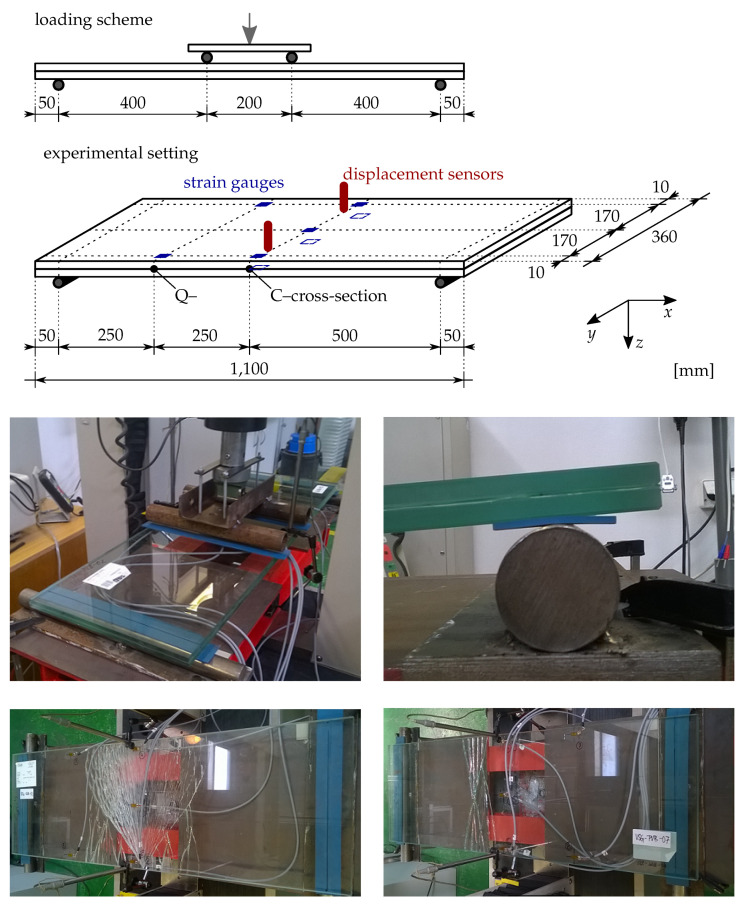
Experimental setup and crack patterns; courtesy of Tomáš Hána from CTU in Prague.

The set of four-point bending tests was performed on five ANG-EVA and five ANG-PVB samples at the Faculty of Civil Engineering, Czech Technical University (CTU), in Prague. The experiments were displacement-controlled with the cross-head speed of the MTS loading device of 0.03 mm/s, [66]. The samples were placed on cylindrical supports and separated with rubber pads (Figure 13). The measured room temperature was 25 ${}^{\circ }C$ The deflections were measured by two linear variable differential transformers (LVDT)—HBM displacement transducers of type W50TK(± 50mm)—positioned in the midspan at the sides of the specimen to check that the experimental setup was symmetric. Additionally, eight strain gauges LY 11-10/120 (HBM, nominal resistance and resistance tolerance 120 Ω ± 0.35%, gauge factor 2.05 ± 1.0%) were attached to the glass surface using adhesive Z 70, five on the upper surface under compression and three on the bottom surface under tension (Figure 13). For the data acquisition, Dewe 2600 from Dewetron was employed, with the sampling frequency of 10 Hz. The strain gauges were placed longitudinally, always two or three gauges through the width of the sample to check if the loading was symmetric and to obtain the distribution of normal stresses along the width of the sample. Three gauges were placed on the glass surface under tension in the area of the largest bending moments to analyse the response during the first stage—until the first glass fracture—and to obtain the failure stresses. Two other sets of strain gauges were placed in two lines on the upper surface under compression to monitor the post-fracture behaviour of the plate. The quarter bridge-type strain gauge arrangement was used to measure the axial strain along the length of the glass (the strain gauge data acquisition device was set as a full bridge with one arm of the bridge being the strain gauge). These strains were converted to stresses using the Young modulus of glass E=70 GPa, [51].

**Figure 14:**
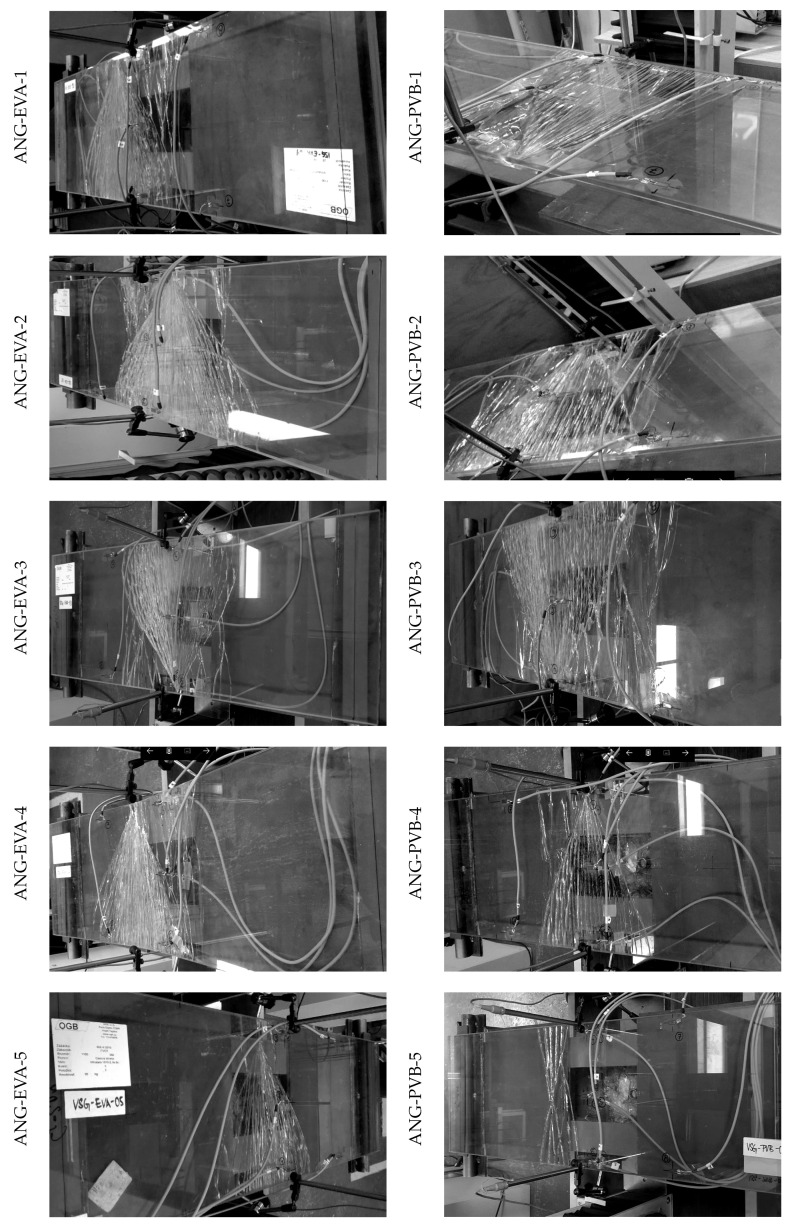
Fracture pattern after first loading for laminated glass samples. Both glass layers were damaged for ANG-EVA-4, whereas the fracture patterns for the other samples correspond only to the fracture of the bottom glass layer; courtesy of Tomáš Hána from CTU in Prague.

Table 8 summarizes the extreme tensile failure stresses for the two types of laminated glass samples independently. These values are utilized for the validation of the numerical solver in the next section. Mostly, the fracture originated from a spot on the edge, see Figure 14.

## 6. Validation of the Phase-Field Model against Experimental Data

In order to assess the behaviour of the phase-field model and the quality of the identified material parameters for both foils, we present a validation of the numerical predictions against the experimentally measured response for the three-layer laminated glass plates.

**Figure 15:**
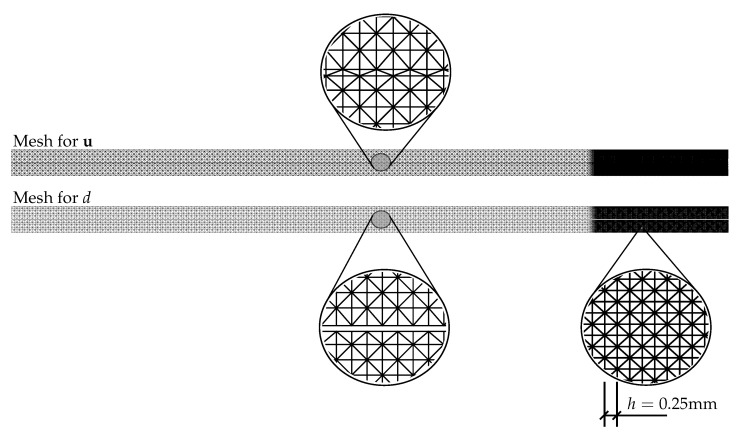
Locally refined discretization for the 2D laminated glass plane-stress model. Phase-field variable *d* is calculated on the sub-mesh in both glass layers only.

Even though the beam formulation is computationally most effective, we selected for the simulation the 2D plane-stress model representing the longitudinal cross-section; see the used mesh in Figure 15. The reasons are that this model takes into account the transverse compression of the interlayer and provides a better way of visualisation of the crack evolution and its final pattern. Moreover, the more expensive plate formulation did not provide additional information and improvement as only one crack was localised near the loading cylinder; recall Section 4.3.

The phase-field sub-problem, i.e., the damage evolution equation, is defined for the glass layers only (recall Figure 15). Therefore, the phase-field is not continuous in the domain, and no crack can evolve in the interlayer. This assumption is in agreement with the displacement-controlled tests when the fractured glass did not break into pieces, as the shards were still connected to the foil; see Figure 13.

Note that this modelling choice implies the homogeneous Neumann conditions on glass layer boundaries; recall Equation (13). Using the same mesh for u and *d* variables would imply the homogeneous Dirichlet boundary conditions at the glass-polymer boundary that result in interfacial crack branching; see, e.g., Figures 21 and 22 in [67] and the accompanying discussion.

### 6.1. Four-Point Bending Tests on Solid Laminated Glass Samples

**Figure 16:**
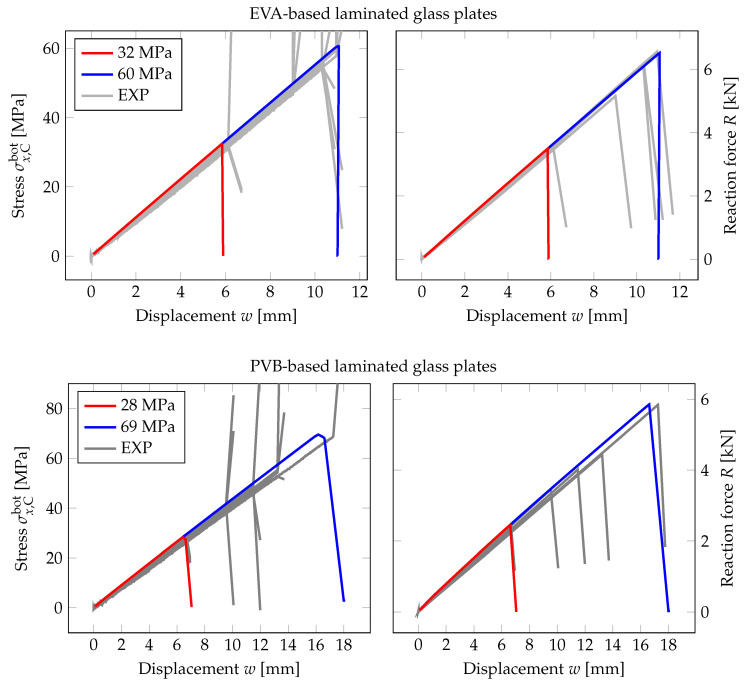
PF-P formulation, anisotropic staggered approach with the spectral-decomposition split, PS model: Evolution of the largest tensile stress at the midpoint and the overall reaction force under the loading points with respect to the midpoint deflection. Experimental data (EXP, grey lines); numerical response for the lowest measured failure stress (red) and for the highest value (blue).

The numerical model is validated against the experimental data for the first loading stage, i.e., until the fracture of one glass layer. For laminated glass, the vertical displacements denoted as *w* in Figure 16 do not correspond to the prescribed positions of the loading head, but to the deflections at the midspan measured during the bending tests; see Figure 13. The fracture energy was set according to Equation (17) using the extreme tensile strengths measured during the experimental testing (Table 8).

For both foils, we achieved a very good agreement with the experimental data for the normal stresses and the overall force reaction of the laminated plate. The numerical prediction was slightly stiffer for some of the laminated glass plates. Except for the error following from the numerical idealisation of the laminated glass response, the deviations in material properties or thicknesses of layers due to the production tolerances could also cause the small overestimation of the laminated glass stiffness. Because the shear coupling was higher for the numerical model, the error in the failure deflection for the failure stress of 69 MPa was about 4%, and the fracture occurred for slightly lower numerical deflection. On the other hand, the numerical and experimental response corresponding to the minimal failure stress of 28 MPa fit well, and the error in deflections was about 1%. For the EVA-based samples, the error in failure deflections was 1% for the largest failure stress of 60 MPa and 3% for the lowest value of 32 MPa.

**Figure 17:**
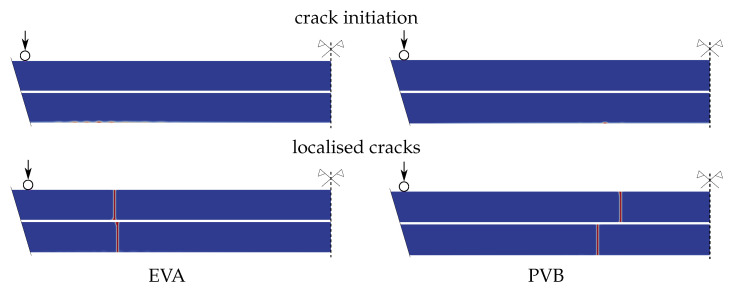
PF-P formulation, anisotropic staggered approach with the spectral-decomposition split, PS model: Crack initiation and localised crack laminated glass samples under four-point bending.

The numerical prediction of the crack evolution obtained by the phase-field fracture model is shown in Figure 17. The first row corresponds to the crack initiation and the second to the position of the localised cracks for both types of laminated glass. Even for a very fine increment of the applied displacement $\Delta w=3\times 10^{-5}$ mm, the cracks appear in both glass layers at the same converged step of the staggered algorithm. For EVA-based samples, the phase-field parameter evolves in a few bumps closer to the loading cylinder, and the cracks localised in glass layers are almost above each other. For the PVB-laminated glass, the bottom crack starts to evolve from only one initial point, and the upper crack is shifted to the centre.

### 6.2. Four-Point Bending Tests on Laminated Glass Samples with One Layer Fractured

During the experimental testing, the bottom glass layer was damaged first for the majority of tested samples, and multiple cracks evolved; see Figure 14. Then, the sample was unloaded, and the fracture of the second ply and so the collapse of the laminated glass sample occurred during the second loading stage.

**Figure 18:**
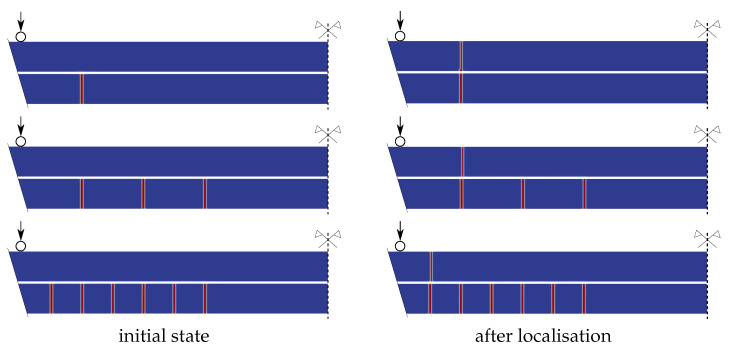
Phase-field variable in pre-cracked laminated glass under four-point bending with a different number of initial cracks in a symmetric half of the sample. Initial crack pattern in bottom glass ply and the final fracture for the EVA-based samples for PF-P formulation, anisotropic staggered approach with the spectral-decomposition split, PS model.

To numerically simulate this second stage of loading and so the residual resistance of the fractured plate, we defined initial crack patterns in the bottom glass layer, consisting of one, three, or six cracks for a half of the sample (Figure 18). The cracks were defined by setting the initial phase-field variable to one with the width of the initial cracks $2l_{c}$. These simulations were performed only for the EVA-based laminated glass samples, and the final crack patterns are shown in Figure 18. Again, only one crack was localised in the upper layer.

**Figure 19:**
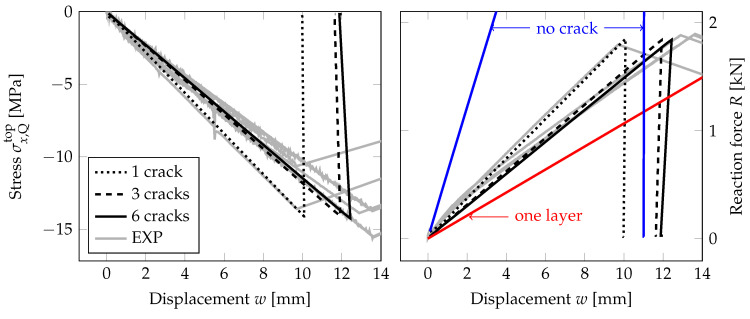
PF-P formulation, anisotropic staggered approach with the spectral-decomposition split, PS model: Evolution of the compressive stress at a quarter of midspan and the overall reaction under the loading points for the mid-span displacement for the second stage of loading and the EVA-based laminated glass plates. Experimental data (EXP, grey lines) and numerical response for one failure stress of 60 MPa (black) with different number of initial cracks.

Figure 19 illustrates the response of the EVA-laminated glass samples with the bottom layer fractured assuming the largest failure stress of 60 MPa. The evolution of compressive stresses on the upper surface at a quarter of the midspan and the overall reaction are plotted for the mid-span displacement. In this case, the tensile stresses are not validated due to the fracture in the bottom glass and the inaccessible bottom surface of the upper glass layer due to the lamination.

The force-displacement diagram in Figure 19 shows the residual resistance of the laminated glass plate with one glass damaged. Two limits bound the stiffness of the samples: the upper limit corresponds to the behaviour of an undamaged laminated glass and the lower bound to the response of a single glass layer. The numerical model with one initial crack in a symmetric half of the bottom glass is stiffer than most of the experimentally measured responses. Therefore, the critical stress is also reached for a lower prescribed displacement. The force-displacement diagram fits well the experimental response for ANG-EVA-5 in Figure 14, where the damage after the first stage of loading is the lowest one for the EVA-based samples, and the sample is stiffer due to the triangular shape of the fracture pattern. Considering more initial cracks in the bottom glass under the largest bending moment results in the slopes of the stress-displacement and force-displacement diagram that match the experimental data better. The difference in the responses of a sample with three or six cracks (on a half of the area under the largest bending) is small. Because we neglected the weight of the fractured glass sample, the numerical post-fracture response does not correspond exactly to that was observed in the experiment.

This analysis revealed that the stiffness of the partially fractured laminated glass can be approximated even with a 2D plane-stress model with initially predefined cracks. The numerical model matched the experimentally measured response very well and provided better estimation than a one-glass-layer limit.

### 6.3. Beam Model for Laminated Glass and the Influence of the Interlayer

**Figure 20:**
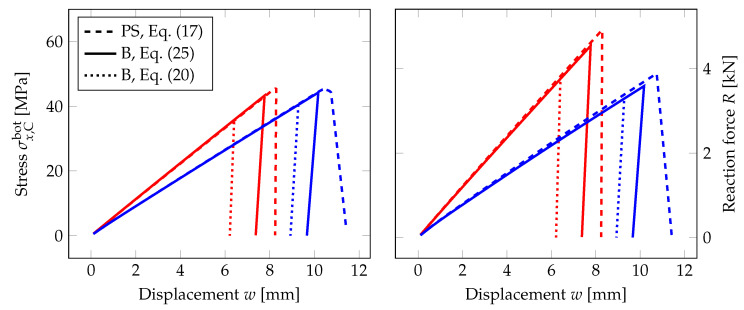
PF-P formulation, anisotropic staggered approach with the spectral-decomposition split: Comparison of the response of laminated glass plates with EVA (red line) or PVB (blue line) using the plane-stress (PS) or beam formulation (B). The evolution of the largest tensile stress at the midpoint and the overall reaction under the loading points for the mid-span displacement. The bending strength of glass is set to 45 MPa.

Finally, Figure 20 compares the numerical response of EVA-based and PVB-based laminated glass samples using the PS model and the B model, corresponding to the plane-stress model of the longitudinal cross-section and to the three-layer beam, respectively. For the loading rate of 0.03 mm/s, the EVA interlayer provides better shear coupling, and the response of the laminated glass sample is stiffer than that of the PVB-samples. Therefore, the critical tensile strength is reached earlier; the fracture occurs for lower deflections, but the resistance of the sample is higher for EVA-based samples.

For laminated glass, the relationship in Equation (20) binding the fracture energy $G_{c}$, the length-scale parameter $l_{c}$, and the critical stress $\sigma_{c}$ cannot be applied for the B model, as it was derived for beams under pure bending only. In reality, normal forces arise from the stress redistribution in both glass layers due to the shear coupling by the interlayer. A simple possibility to overcome this problem is to drive the phase-field evolution in Equation (13) by the positive elastic energy density on the surface in tension, as the following shows.

As the B-model assumes a constant value of the phase-field variable through the thickness, the governing equation of damage evolution (13) is integrated over the cross-section to obtain:

(24) $$\begin{array}{c} \frac{A}{c_{\alpha }}\left(\frac{d\alpha (d)}{dd}-2l_{c}^{2}\frac{d^{2}d\left(x\right)}{dx^{2}}\right)=-\frac{l_{c}}{G_{c}}\frac{dg(d)}{dd}\int_{A}\psi_{e}^{+}\;dA, \end{array}$$

where *A* is the transverse cross-sectional area. Therefore, the phase-field evolution is driven by the stored energy density integrated over the cross-sectional area under tension instead of the extreme value at the surface fibres. To modify this behaviour, we replace the right-hand side integral with:

(25) $$\begin{array}{c} \int_{A}\psi_{e}^{+}\;dA\approx \frac{EA}{2}\max\left({\langle \varepsilon \left(x,h/2\right)\rangle }^{2},{\langle \varepsilon \left(x,-h/2\right)\rangle }^{2}\right), \end{array}$$

where the strain $\varepsilon \left(x,z\right)=\frac{du}{dx}+z\frac{d\varphi }{dx}$ is obtained from the centerline horizontal displacement *u* and cross-sectional rotation φ. This modification of the driving force is performed for each glass layer separately, so a crack evolves through the layer once a critical value is reached on one of its surfaces.

The comparison in Figure 20 shows that the through-thickness compression of the interlayer does not significantly effect the response. The differences in the slopes of the force-displacement diagram for both formulations are negligible. The glass fracture occurs for the beam formulation earlier as the criterion is set according to the bottom surface, but the differences are considerable smaller than for the parametric studies performed in Section 4.

## 7. Conclusions

Using the phase-field fracture models, we studied the brittle response of monolithic and laminated glass plates under bending with the goal:

- to compare three different modelling strategies based on the phase-field fracture formulations and analyse the extent of the nonlinear part of the expected linear pre-fracture response,
- to discuss the possible dimensional reduction of the problem together with the setting of the length-scale parameter and adjusted fracture energy of glass,
- to illustrate how the fracture for the four-point bending tests initiated from glass edges could be enforced,
- to predict the response of laminated glass with one layer fractured.

The main contributions of the presented study are:

- For thin plates under bending, the nonlinear response due to the damage before the crack localisation can be significant for some phase-field formulations. This effect can lead to a considerable overestimation of the response.
- The fracture energy of soda-lime-silica glass results in a very small length-scale parameter. For numerical purposes, the fracture energy should be determined concerning the applied dimensional reduction, loading type, and the value of the length-scale parameter compared with the thickness of the structure under bending. Then, a consistent value of the tensile strength corresponds to the glass fracture.
- Two possible ways of the fracture energy scaling were introduced in this study for beam or plate models. For a one-layer monolith under bending, the scaling relations were derived from the evolution equation using a spatially homogeneous solution. For multi-layer plates, the phase-field evolution can be driven by a positive energy density on a surface in tension, as suggested in this study.
- For the plate model, the strength has to be reduced in a band or area of sufficient dimensions and not only at nodes directly on edges to reproduce cracking from edges.
- The comparison showed that the numerical model provides a very good agreement with the measured stresses and resistance of laminated glass, even though only one or two cracks were localised for all discretisations using the quasi-static solver, whereas multiple cracks evolved during the experiment. The differences in failure deflections and stresses were under 5%, and these errors were also effected by the fact that nominal dimensions were employed and the Young modulus and Poisson ratio of glass were taken from standards, as is common in design practice.
- The stiffness and resistance of the partially fractured laminated glass can be approximated with a 2D plane-stress model with initially predefined cracks. The model matched the experiment very well and provided much better estimation than a one-glass-layer limit.
- For quasi-static loading of laminated glass, the presented examples also validated the time-/temperature-dependent material properties of the ethylene-vinyl acetate or polyvinyl butyral interlayers derived recently by the authors in [28].

To achieve the propagation of multiple cracks, the model has to be extended by dynamic effects and/or the glass strength should be considered randomly distributed across the glass layers. Currently, we are working on the extension of the model to incorporate the effect of the stochastic strength of glass.

## Figures and Tables

**Table 1:** Strain energy decompositions: volumetric-deviatoric split (VD) and spectral decomposition (SD), where *K* stands for the bulk modulus and λ,μ for Lame’s coefficients. $\varepsilon_{D}$ is the deviatoric part of the strain tensor. The Macaulay brackets are defined as $\langle a\rangle =\left(a+|a|\right)/2$, and the positive/negative components $\varepsilon^{\pm }=\sum_{i}\pm \langle \pm \varepsilon_{i}\rangle p_{i}⊗p_{i}$ follow from the principal strains $\varepsilon_{i}$ and the dyadic products of the eigenvectors $p_{i}$ of ε.

Split	$\psi_{e}^{+}\left(\varepsilon \right)$	$\psi_{e}^{-}\left(\varepsilon \right)$
VD	$K/2{\langle \mathrm{tr}\left(\varepsilon \right)\rangle }^{2}+\mu \varepsilon_{D}:\varepsilon_{D}$	$K/2{\langle -\mathrm{tr}\left(\varepsilon \right)\rangle }^{2}$
SD	$\lambda /2{\langle \mathrm{tr}\left(\varepsilon \right)\rangle }^{2}+\mu \varepsilon^{+}:\varepsilon^{+}$	$\lambda /2{\langle -\mathrm{tr}\left(\varepsilon \right)\rangle }^{2}+\mu \varepsilon^{-}:\varepsilon^{-}$

**Table 2:** Phase-field formulations and corresponding functions and parameters. For the PF-M model, the Macaulay brackets are defined as $\langle a\rangle =\left(a+|a|\right)/2$. $\sigma_{c}$ is a critical fracture stress corresponding to the tensile strength, and ${\bar{\sigma }}_{i}$ are the principal components of the effective stress tensor.

Model	α(d)	$c_{\alpha }$	g(d)	Criterion	$\tilde{Y}$
PF-B	$d^{2}$	2	${\left(1-d\right)}^{2}$	energetic	$\frac{2\psi_{e}^{+}\left(\varepsilon (u)\right)}{G_{c}/l_{c}}$
PF-M	$d^{2}$	2	${\left(1-d\right)}^{2}$	Rankine-based (stress)	$\langle \frac{1}{\sigma_{c}^{2}}\sum_{i}{\langle {\bar{\sigma }}_{i}\rangle }^{2}-1\rangle$
PF-P	*d*	8/3	${\left(1-d\right)}^{2}$	energetic	$\frac{2\psi_{e}^{+}\left(\varepsilon (u)\right)}{G_{c}/l_{c}}$

**Table 3:** Elastic material parameters and tensile strength of glass [51].

Glass			
Young’s modulus of elasticity	*E*	70	GPa
Poisson’s ratio	ν	0.22	–
Tensile strength	$\sigma_{c}$	45	MPa

**Table 4:** Link between the fracture energy $G_{c}$ and the length-scale parameter $l_{c}$ seen as a material or numerical parameter using the Young modulus E=70 GPa and the tensile strength for annealed glass $\sigma_{c}=45$ MPa from [51].

	$G_{c}$ [53]	$l_{c}=\frac{27}{256}\frac{EG_{c}}{\sigma_{c}^{2}}$	$l_{c}=\frac{3}{8}\frac{EG_{c}}{\sigma_{c}^{2}}$	$G_{c}=\frac{256}{27}\frac{\sigma_{c}^{2}l_{c}}{E}$	$G_{c}=\frac{8}{3}\frac{\sigma_{c}^{2}l_{c}}{E}$
	(J·m${}^{-2}$)	(mm)	(mm)	(J·m${}^{-2}$)	(J·m${}^{-2}$)
material parameter	4	0.010	0.036
numerical parameter		3	3	823	231

**Table 5:** Material composition of the layers in the tested specimens.

Name	Glass	Interlayer	Samples
ANG-EVA	annealed	ethylene-vinyl acetate	5
ANG-PVB	annealed	polyvinyl butyral	5

**Table 6:** Prony series for the generalised Maxwell model with the relaxation times $\tau_{p}$ and corresponding shear moduli $G_{p}$ for the reference temperature $T_{0}=20{\;}^{\circ }C$ and the long-term moduli $G_{\infty }=682.18$ kPa for EVALAM 80-120 (EVA) and $G_{\infty }=232.26$ kPa for TROSIFOL BG R20 (PVB), [28].

	EVA	PVB		EVA	PVB
$\tau_{p}$ (s)	$G_{p}$ (kPa)	$G_{p}$ (kPa)	$\tau_{p}$ (s)	$G_{p}$ (kPa)	$G_{p}$ (kPa)
$10^{-9}$	6933.9	–	$10^{2}$	445.1	587.2
$10^{-8}$	3898.6	–	$10^{3}$	300.1	258.0
$10^{-7}$	2289.2	–	$10^{4}$	401.6	63.8
$10^{-6}$	1672.7	–	$10^{5}$	348.1	168.4
$10^{-5}$	761.6	1,782,124.2	$10^{6}$	111.6	–
$10^{-4}$	2401.0	519,208.7	$10^{7}$	127.2	–
$10^{-3}$	65.2	546,176.8	$10^{8}$	137.8	–
$10^{-2}$	248.0	216,893.2	$10^{9}$	50.5	–
$10^{-1}$	575.6	13,618.3	$10^{10}$	322.9	–
$10^{0}$	56.3	4988.3	$10^{11}$	100.0	–
$10^{1}$	188.6	1663.8	$10^{12}$	199.9	–

**Table 7:** Parameters for the time-temperature superposition using the William–Landel–Ferry equation [65].

		EVA	PVB	
Reference temperature	$T_{0}$	20	20	${}^{\circ }C$
Parameters	$C_{1}$	339.102	8.635	–
	$C_{2}$	1185.816	42.422	${}^{\circ }C$
Shift parameter	$\alpha_{T}(T=25$${}^{\circ }C$)	0.03769	0.1229	–

**Table 8:** Extreme tensile failure stresses on the bottom surface of laminated glass under four-point bending.

Samples	Min Failure Stress (MPa)	Max Failure Stress (MPa)
ANG-EVA	32	60
ANG-PVB	28	69
